# The smallest space miners: principles of space biomining

**DOI:** 10.1007/s00792-021-01253-w

**Published:** 2022-01-06

**Authors:** Rosa Santomartino, Luis Zea, Charles S. Cockell

**Affiliations:** 1 UK Centre for Astrobiology, School of Physics and Astronomy, University of Edinburgh, Edinburgh, EH9 3FD UK; 2 BioServe Space Technologies, University of Colorado Boulder, Boulder, CO USA

**Keywords:** Space biomining, ISRU, Space bioleaching, BLSS, Space microbiology, Bioremediation, Space sustainability

## Abstract

As we aim to expand human presence in space, we need to find viable approaches to achieve independence from terrestrial resources. Space biomining of the Moon, Mars and asteroids has been indicated as one of the promising approaches to achieve in-situ resource utilization by the main space agencies. Structural and expensive metals, essential mineral nutrients, water, oxygen and volatiles could be potentially extracted from extraterrestrial regolith and rocks using microbial-based biotechnologies. The use of bioleaching microorganisms could also be applied to space bioremediation, recycling of waste and to reinforce regenerative life support systems. However, the science around space biomining is still young. Relevant differences between terrestrial and extraterrestrial conditions exist, including the rock types and ores available for mining, and a direct application of established terrestrial biomining techniques may not be a possibility. It is, therefore, necessary to invest in terrestrial and space-based research of specific methods for space applications to learn the effects of space conditions on biomining and bioremediation, expand our knowledge on organotrophic and community-based bioleaching mechanisms, as well as on anaerobic biomining, and investigate the use of synthetic biology to overcome limitations posed by the space environments.

## Introduction

As the goal for human space exploration moves farther from Earth, relying on a constant resupply of resources from Earth becomes less viable. In-situ resource utilization (ISRU) approaches have the aim of enabling sustainable exploration and settlement of space, by mitigating the need of importing goods and materials from Earth (NASA-ASEE 1992; Anand et al. 2012; Montague et al. 2012; Menezes et al. 2015b; Verseux et al. 2016). Microbial-based biotechnologies and biomanufacturing approaches have demonstrated their high efficacy in several applications and may become a key component of future ISRU strategies (Brown et al. 2008; Slenzka and Kempf 2010; Montague et al. 2012; Verseux et al. 2016; Nangle et al. 2020; Volponi and Lasseur 2020; Berliner et al. 2021; Castelein et al. 2021; Keller et al. 2021). Minerals and metals will be necessary resources for construction, infrastructure and manufacturing. Water, oxygen, volatiles, essential nutrients for human nutrition and soil fertilization, are all elements which will be essential to establish self-sufficient settlements, and that could be obtained from extraterrestrial rocks (Cockell 2011). Mining of these resources will therefore be a priority for an extraterrestrial settlement.

Microorganisms can be applied to these needs, extracting useful elements by a technique called biomining or bioleaching. Currently on Earth around 20–25% of copper and 5% of gold are extracted using biomining (Johnson et al. 2013). Bioleaching can provide economic and environmental advantages compared to other methods (Srichandan et al. 2019) and can be used as a complementary technique to extract trace metals from mine waste, or recover low-grade ores which cannot be treated by conventional methods (Bosecker 1997). Mining processes produce an enormous amount of waste low-grade rock material and mill tailings, which often still contain useful trace elements that cannot be further recovered by traditional methods. However, these can be accessible to small microbial cells, as an effect of specific types of metabolism (Blowes et al. 2003; Schippers et al. 2013; Johnson 2014; Jerez 2017a). With the same principle, bioleaching techniques can be applied to the recycling of useful metals from secondary solid waste (Schippers et al. 2013; Johnson 2014; Jerez 2017b). A good example is provided by the recovery of rare earth elements (REEs, Reed et al. 2016) or copper from electronic waste (Srichandan et al. 2019).

Many advantages of biomining over traditional techniques on Earth also apply to space. For instance, biomining generally requires lower energy compared to traditional mining (Schippers et al. 2013), which will be an advantage in space, where resources are limited and many engineering constraints may be in place. Nevertheless, space conditions and available rock materials are different from those present on Earth and which are commonly used in terrestrial biomining. On one hand, traditional autotrophic and chemolithotrophic microbes used for terrestrial biomining do not need to rely on organic compounds as nutrients, which are very scarce in extraterrestrial environments, to grow (Johansson 1992; Fahrion et al. 2021). On the other hand, rocks present on celestial bodies of mining interest have very diverse compositions. In some cases (e.g., the Moon and some asteroids) sulphide-containing minerals are scarce compared to ore-grade sulphide minerals which are the focus of terrestrial biomining (Lewis 1992; Anand et al. 2012; Righter et al. 2017; Steenstra et al. 2018, 2020; Brounce et al. 2019, 2020). This poses restrictions on the possibility of directly transferring our current knowledge on bioleaching, which is mainly based on chemolithoautotrophic iron- and/or sulphur-oxidising microorganisms, to space.

This review explores the topic of space biomining, discussing the main principles and feasibility, and analyses possible applications. We discuss limitations and highlight that, considering the materials available in space, it is necessary to expand our knowledge on organotrophic, cyanobacteria and community-based bioleaching mechanisms. Finally, we propose to consider the addition of biomining compartments to supply basic chemical requirements for Biological Life-Support Systems (BLSS), and vice versa.

## Space biomining principles

When talking about space biomining for ISRU, the following three main questions have to be addressed: (1) What elements do we need to obtain for a given application? (2) Where can we find them? (3) Which mining strategy would work best (this could exclude biomining in some cases)? We will try to address these questions, to the best of our knowledge, in this section. Answering these questions can be complex, as it involves an interdisciplinary knowledge of the space conditions present on the planetary body we want to mine, the type of rocks and mineral compositions available (including their intrinsic leachability and toxicity), the engineering technologies and biotechnologies required, in-space operational approach (a thorough discussion on the requirements for life support systems and ISRU for space exploration, although focused on cyanobacteria-based structures, has been reported in Keller et al. 2021), and the appropriate microorganism(s) and its metabolism(s) (Averesch 2021). The situation becomes even more complicated when discussing asteroid biomining, given that the mined resources would likely not be used in situ, therefore requiring additional transport to the location of utilization (see Sect. 4.3).

To answer the first question, elements of high interest from the space biomining perspective vary depending on the aim (Cockell 2011; Raafat et al. 2013). Generally speaking, elements and molecules of interest for the establishment of a human settlements are water, molecular oxygen, essential mineral nutrients (e.g., potassium, sodium, calcium, phosphorus, sulphur), gaseous volatiles (e.g., hydrogen, carbon, nitrogen, helium), structural metals such as iron, copper, nickel and vanadium, and elements useful for electronic devices such as silicon and REEs (Menezes et al. 2015a, b). Some of these, especially volatiles, could be used to produce fuel or water (such as hydrogen, Klas et al. 2015). Of great economic interest are also elements as platinum group elements (PGE), gold, and silver, which are currently expensive. This list is not intended to be complete. It must be noted that many rocks in the Solar System would be regarded as low-grade ores, in respect to those considered suitable for mining on Earth (Cockell and Santomartino, in press). However, biomining on Earth is often used to process mine waste, dumps and tailings which cannot be easily treated by conventional methods (Bosecker 1997; Mishra and Rhee 2014; Klas et al. 2015).

Growth, and in some cases bioleaching, of diverse microorganisms on extraterrestrial rocks or simulants have been demonstrated (Gronstal et al. 2009; Olsson-Francis and Cockell 2010; Kölbl et al. 2017; Metzger et al. 2020; Volger et al. 2020; Milojevic et al. 2021), suggesting that, with the appropriate selection of species or consortia and conditions, microbial interaction with extraterrestrial substrate is possible. Moreover, space biomining experiments have been already performed in space. These include BioRock (Cockell et al. 2020, 2021; Santomartino et al. 2020) and BioAsteroid (2020/2021, data analysis in preparation), both performed onboard the International Space Station (ISS) and a preparative mission for BioRock (Byloos et al. 2017). Although not intended as fully scaled-up biomining experiments, they first demonstrated the feasibility of biomining in space using heterotrophic microorganisms (bacteria and fungi), and basalt or meteorite as substrates.

### Space conditions and effects on biomining

Environments outside Earth show a variety of conditions which differ from those present on Earth’s surface. Their effects on microbial behaviour at different levels (e.g., growth, survival, resistance, virulence, biofilm formation) have been widely reported (reviewed in Horneck et al. 2010; Senatore et al. 2018), and still there is much yet to be understood (Cockell 2021). Space can be considered an umbrella term which includes a variety of environments, each one with its own characteristics. For the aim of this review, we will report the following conditions present in three different space environments of relevance for the space biomining perspective: Mars, the Moon and asteroids (Table 1). On asteroids, given their small mass, we can generally assume conditions equivalent to that of spaceflight beyond Earth’s Van Allen belts, as described below.

**Table 1:** Conditions on Moon, Mars and asteroids that could influence space biomining, compared to those on Earth

Condition	Planetary body				
	Mars	Moon		Asteroids	Earth
Gravity	0.38×*g*	0.16×*g*		Micro to centigravity	1×*g*
Ionizing radiations	~ 20 µGy/hour (GCR, SEP)	~ 13.2 µGy/hour (GCR, SEP)		(GCR, SEP)	0.034–0.114 µGy/hour (Baumstark-Khan and Facius 2002)
Atmosphere composition	95% CO_2_, 2.8% N_2_, 2.1% Ar, trace gases	Negligible		Absent	78% N_2_, 20.9% O_2_, 0.9% Ar, 0.04% CO_2_, trace gases
Pressure	6.1 mbar	3 × 10^–15^ bar		Vacuum	1 bar (at sea level)
Temperature	− 153 °C to + 20 °C	− 178 °C to + 124 °C		Depends on the asteroid. Generally < − 20 °C	− 89.2 °C (Turner et al. 2009) to + 56.7 °C (El Fadli et al. 2013)
Rock composition	Basalt containing plagiocase, pyroxene, olivine and sulphate minerals	Basalt, anorthosite and breccia. Minerals include plagioclase, pyroxene, olivine, ilmenite, spinel and others. KREEP rocks		Depending on the type. Can contain water, volatiles, rich elements (PGE), common metals, organic compounds	Primarily basalt and granite, a vast diversity of igneous sedimentary and metamorphic rocks

When not stated in the table, see the main text for references. *GCR* galactic cosmic rays, *SEP* Solar energetic particles

All the conditions described here assume that the biomining operation has access to liquid water, an essential requirement for life (Cockell 2021). Water must either be obtained on the body of interest or imported. Although this is critical for biomining operations, here we focus on the other environmental conditions within a biomining reactor that influence growth within a liquid environment.

i. *Gravity* Gravity is proportional to the mass of the celestial body under examination. As a consequence, gravities at the surface of Mars, the Moon and asteroids are lower than on Earth (9.8 m/s^2^, or 1×*g*). Mars has a gravity regimen of 3.7 m/s^2^ (~ 0.38×*g*), the Moon 1.62 m/s^2^ (~ 0.16×*g*). Microgravity (µ*g* or ~ 10^–6^×*g*) is the gravity condition generally experienced in a spacecraft in a free trajectory (either in transit from one celestial body to another, or in orbit around one; Horneck et al. 2010; Zea et al. 2017; Huang et al. 2018). Gravitational levels at the surface of an asteroid depend on the mass and the irregularity of its shape. While for more massive objects such as Ceres (0.27 m/s^2^, or ~ 0.03×*g*), now considered a dwarf planet, gravity may be slightly higher (centigravity, in fact), the majority of asteroids are expected to have a gravity regimen similar to microgravity (Klas et al. 2015). It has been reported that cells with a diameter of less than 10 µm, which is the case for bacteria, archaea and many fungi, are too small to be directly affected by gravity or the lack thereof (Pollard 1965; Horneck et al. 2010; Zea et al. 2017). Instead, the responses observed are likely to be the indirect effects of altered extracellular environment where mass transport to and from the cell is limited to Brownian motion, limiting nutrient availability and resulting in the build-up of metabolic by-products with respect to 1×*g* (Bosecker 1997; Zea 2015; Zea et al. 2016). However, microbial structures, namely biofilms, can be orders of magnitude larger. While biofilm growth is well known to occur on spacecraft components during spaceflight (McLean et al. 2001; Zea et al. 2020), controlled experiments have not shown consistent results, as increase, decrease, and no changes on biofilms grown in microgravity with respect to controls on Earth have been reported (reviewed in Zea et al. 2018). However, the effects of gravity on microbe-mineral interaction have not been characterized yet and should be further investigated. BioRock, a recent space biomining experiment mentioned above, showed no difference in final cell concentrations for three biomining bacterial species after 3 weeks of growth under terrestrial, Martian and micro-gravity (Santomartino et al. 2020). This result suggests that space microbial biotechnologies, including biomining, could be indifferent to gravitational conditions. Nevertheless, various hardware settings and reactor volumes may lead to divergent results, and other microbial species could react differently. Furthermore, processes that require gravity (e.g. sedimentation) will be impacted by changes in gravitational conditions.
ii. *Radiation* Earth’s magnetosphere and atmosphere protect us on the surface from most of the radiation environment experienced in space. Beyond the Van Allen belts (Prölss 2004), usually referred to as ‘deep space’, a radiation environment, mostly consisting of (i) galactic cosmic rays (GCR) and (ii) Solar energetic particles (SEP), exists (Zeitlin et al. 2013; Zhang et al. 2020). In the case of the lunar surface, neutrons as secondary particles arising from the interaction of GCRs and SEP’s and the regolith may have an impact on biological systems that is yet unknown (Horneck et al. 2010; Klas et al. 2015; Zhang et al. 2020). Hence, biomining operations beyond the Van Allen belts (i.e., on the Moon, Mars, and asteroids) need to consider the higher radiation environment. A wide literature studied microbial response and resistance to high dose of space radiations, including long-term exposure experiments outside the ISS (Horneck et al. 2012). Many of these studies focused on resistance and survival after exposure in a dried or dormant state. Among the microorganisms that showed a higher resistance to space radiations we can find *Chrooccocidiopsis* (Cockell et al. 2005; Billi et al. 2019), *Bacillus subtilis* (Moeller et al. 2014; Ulrich et al. 2018; Nicholson and Ricco 2020) and *Deinococcus radiodurans* (Mattimore and Battista 1996; Minton 1996), and some fungi as *Aspergillus niger* (Cortesão et al. 2020). Many of these have been also tested for their biomining (Brandl et al. 1999; Rozas et al. 2017; Faraji et al. 2018; Giese et al. 2019) or biosorption (Liu et al. 2012; Jaafar et al. 2015) capacity, suggesting a possible role for biomining in space. It must be noted that some of the molecular mechanisms of radiation (and related stresses) tolerance require the entrance of the cell in a dormant state (e.g., endospore formation, desiccation). These may not be useful in a context of space biomining, in which metabolically active cells are required. Further research is, therefore, necessary to link these two fields of study, in order to understand the most suitable microorganisms or mechanisms for long-term biomining operations.
iii. *Atmosphere composition and pressure* Mars has a thin atmosphere, composed of approximately 95% of CO_2_, 2.8% of N_2_, 2,1% Ar, trace gases including oxygen (0.13%) and hydrogen (Franz et al. 2017), and dust, with an average pressure at the surface of 6.1 mbar (Haberle 2015). Lunar atmosphere (composed of argon, helium, neon, sodium, potassium, hydrogen, trace elements and dust), with a pressure at the surface of 3 × 10^–15^ bar and an average abundance at the surface of 2 × 10^5^ particles/cm^3^, is considered negligible (Stern 1999; Lawson et al. 2005). Asteroids, including Ceres, possess no atmosphere (Hughes 1994, Table 1). From a biomining perspective, the absence of oxygen constitutes a clear problem for those applications requiring aerobic microorganisms. The majority of bioleaching mechanisms elucidated so far occur in aerobic conditions. Nevertheless, anaerobic bioleaching has been demonstrated for some bacteria, although the mechanisms are not fully understood yet (Singh and Cameotra 2015). In anaerobic reductive dissolution, Fe^3+^ is used as electron acceptor. *A. ferrooxidans*, for instance, can catalyse the reduction of Fe^3+^ under anaerobic and acidic conditions, coupled with oxidation of a *c*-type cytochrome (Ohmura et al. 2002; Hallberg et al. 2011; Johnson et al. 2013; Singh and Cameotra 2015; Marrero et al. 2020). *Shewanella onediensis*, a facultative anaerobe, could extract iron from lunar and Martian simulants under aerobic and anaerobic conditions (Castelein et al. 2021). Microorganisms with alternative types of metabolism, or bioengineering approaches, may also be used. More problematic may be the low pressure or, in the case of the Moon and asteroids, vacuum. Pressure is one of the parameters that can profoundly influence microbial proliferation, mostly due to desiccation and reduced gas concentration, and Martian atmospheric pressure is already too low to support most microbial growth (Schwendner and Schuerger 2020; Verseux 2020). The current lowest pressure recorded for microbial growth is 0.7 kPa (7 mbar; Schuerger and Nicholson 2016). Nevertheless, low-pressure vacuum is used in biotechnologies, including biomining (Gnida 2020), and a bioreactor using low-pressure Mars-similar gas composition was successfully developed for cyanobacterial growth (Verseux et al. 2021). This suggests that, with the appropriate adaptation and engineering, challenges posed by extraterrestrial atmospheric composition and pressure could be overcome not to limit biomining applications. Low pressure could, indeed, reduce the engineering requirements of bioreactors.
iv. *Temperature* Temperature can influence not only microbial growth, but also the availability of liquid water for medium growth. Considering the prohibitive space temperatures and variations, discussed below, any space biotechnological application will need to use temperature-controlled bioreactors. However, temperatures of biological-based processes are generally lower than those using chemical or physical processes (Johnson 2014), and bioengineering and synthetic biology approaches could improve biomining capacity at low temperatures in order to decrease energy requirements. Martian surface temperature undergo considerable variations, leading to values varying from − 153 to + 20 °C (Murphy et al. 1990; Haberle 2015). Due to the negligible atmosphere, surface temperature on the Moon varies between − 178 °C and + 124 °C (Williams et al. 2017). Asteroid surface temperatures depend on the distance from the Sun as well as from local differences in composition and topographic features, and they can vary largely among asteroids. On Ceres, surface temperatures oscillates between − 143 and − 73 °C, and similar conditions can be expected on smaller asteroids. For example, the highest surface temperature on asteroid 21 Lutetia, measured by Visible, InfraRed, and Thermal Imaging Spectrometer (VIRTIS) on Rosetta, was − 28 °C (Coradini et al. 2011), and the surface temperature of Ryugu, measured by the Japanese Hayabusa2 orbiter, varies between − 50 °C and + 60 °C (Okada et al. 2020). In other words, on all extraterrestrial bodies known, temperatures tend to generally exceed the known limits to life, implying that biomining operations will need to be conducted under strict temperature control.
v. *Rock composition, regolith and minerals* Regolith and rock composition need to be taken into account when deciding which biomining microorganism or technique to use in a given region of a planetary body, to extract the desired element(s), especially considering that they could also contain toxic compounds, for instance perchlorates (Hecht et al. 2009; Linnarsson et al. 2012; Kounaves et al. 2014; Wadsworth and Cockell 2017). Fine regolith is particularly interesting from the mining perspective, as extracting elements from it would spare the energy required for rock crushing (Carter 1992). The composition of Mars and Moon crusts have been highly studied and characterized. This allowed the preparation of several Martian and lunar analogue materials, which can be used to test if they could provide good substrates for microbial growth and elemental extraction (Olsson-Francis and Cockell 2010). A variety of meteorites are available to study asteroid composition, and microbial growth on meteorites has been demonstrated (Tait et al. 2017; Milojevic et al. 2019).
  Our knowledge on Martian rock composition comes from landers, orbiting spacecrafts and Martian meteorite data. Martian bulk crust is mostly igneous and basaltic (McSween et al. 2009; Ehlmann and Edwards 2014; Yoshizaki and McDonough 2020), with a high level of oxidized iron and magnesium (Clark 1993; Rieder et al. 1997; Yen et al. 2005; McSween et al. 2009). Major minerals present in the Martian crust are silicates such as plagioclase, pyroxene and olivine (Ehlmann and Edwards 2014). FeO is also particularly abundant (Taylor 2013). Previous studies reported a high sulphur composition in Martian surface, mostly in the form of sulphate minerals (Greenwood et al. 2000; Franz et al. 2019). Clay, carbonate and sulphate deposits have been found, and calcium, iron and magnesium sulphate minerals have been identified from landers and orbital data (Ehlmann and Edwards 2014). However, recent estimations on sulphur abundance in Martian interiors suggest that sulphur and volatile content could be much less than previously indicated (Wang and Becker 2017; Franz et al. 2019; Yoshizaki and McDonough 2020), and sulphur has been found in small scale in certain zones of Martian surface (Ehlmann and dwards 2014). Zinc content is quite high while copper may be less present than on Earth, although copper-rich deposits could exist in specific Martian regions (Payré et al. 2019).
  Lunar composition is largely silica-saturated basalts, anorthosite and breccia (Jolliff 2008); however, specific compositions differ around the crust, and between highlands and maria (Jolliff et al. 2000; Arai et al. 2008). Therefore, the relative abundance of minerals in lunar rocks or regolith depends on the location (Sivakumar et al. 2017). They include plagioclase, pyroxene, olivine, ilmenite, spinel and less abundant minerals (Meyer 2003; Ohtake et al. 2009; Anand et al. 2012; Sivakumar et al. 2017), and they are anhydrous and reduced (Jolliff 2008). Interestingly, new mineral species have been identified on the Moon, for instance tranquillityite (Jolliff 2008). Sulphide-bearing apatite was also discovered in lunar rocks, although apatite is present only as accessory phases (Brounce et al. 2019, 2020), as well as sulphides (Anand et al. 2012). Therefore, sulphur appears to be less abundant on the Moon compared to Mars and some asteroids (Righter et al. 2017; Steenstra et al. 2018). Lunar regolith follows a similar composition dominated by aluminosilicate basic rocks (anorthosites, nortic anorthosites, gabbroic anorthosite; Ohtake et al. 2009), with particle size between 10 nm and 100 µm. Mining fine lunar regolith has been proposed as particularly interesting from the mining perspective, because it would not require rock crushing (Carter 1992). Moreover, lunar regolith presents solar wind implanted volatiles of great interest for ISRU, such as hydrogen, nitrogen, carbon and helium (Anand et al. 2012). The presence of helium has attracted interest, as initial calculations estimated a possible cost-efficient transport to Earth as a source of clean energy (Wittenberg et al. 1986). However, more recent evaluations are less positive (Carter 1992; Anand et al. 2012). Particularly promising from a biomining perspective is also a region named Procellarum KREEP terrane (PKT), characterized by high percentage of potassium, REEs, phosphorus and thorium (Taylor et al. 2006).
  Owing to the large number of different types of asteroids, specific cases should be considered when discussing asteroid biomining. Meteorites are made of material from diverse planetary bodies, including asteroids (Housen et al. 1979), but asteroid composition varies widely depending on the type. A complete description of asteroidal composition is beyond the scope of this review; however, information comes largely from the analysis of available meteorites, which are mainly chondrites (Lewis 1992; Yoshizaki and McDonough 2021). Meteorites can be divided into stony, stony-iron and iron. Stony chondritic meteorites are a class of undifferentiated meteorites that are highly interesting from the biomining perspective and compose the large part of those that fall on Earth. Composition varies largely between types. Carbonaceous chondrites (C chondrites) are particularly rich in organic compounds, nitrogen and sulphur; enstatite chondrites (E chondrites) are highly reduced and contain silicon, iron, nickel and sulphides; the most abundant group falls in the intermediate categories of H, L and LL chondrites, with free metal elements, decreasing iron content and a lower presence of sulphur in respect to the former two (Lewis 1992). Water, volatiles, organic compounds, elements as the platinum group elements (PGE: platinum, osmium, ruthenium, rhodium, palladium and iridium) and more common metals such as iron or nickel can be present, in quantities depending on the asteroid type (Klas et al. 2015; Steenstra et al. 2018, 2020). Sulphur depletion on some asteroids and meteorite surfaces has been documented, as a consequence of space weathering of iron sulphides (Matsumoto et al. 2020). To mention a few notable asteroid examples, 21 Lutetia has a metal-rich composition with mafic silicate minerals poor in iron and hydrated minerals (Coradini et al. 2011), Ceres is rich in hydrated minerals, ammoniated phyllosilicates and carbonates, salts and organic material (De Sanctis et al. 2017). Similarly, 24 Themis (Campins et al. 2010) and Bennu (Lauretta et al. 2019) have water and organic compounds on their surface. Samples from Itokawa (S-type asteroid), obtained in the Hayabusa mission, revealed not only a composition similar to LL-chondrites, but also provided insights into regolith composition and properties (Tsuchiyama et al. 2011).We will shortly have data from the JAXA Hayabusa2 and the NASA OSIRIS-Rex missions on the returning samples from Ryugu and Bennu, respectively.
  Crucial within the context of all these materials is that, when they are added to a liquid water environment for biomining, they do not result in water activity levels or toxicity that makes them uninhabitable for biomining organisms. Unless the parent material contains high concentrations of salts (e.g., NaCl) there is no a priori reason why any of the materials discussed above should result in unsuitable water activities. The potential toxicity caused by ions (e.g., heavy metals) will depend on the material and the organisms used, but clearly this must be considered for any given combination of minerals and biomining organisms proposed (Averesch 2021).

### Terrestrial biomining mechanisms and space applicability

Bioleaching can be performed by chemolithotrophic and organotrophic microorganisms (Asghari et al. 2013; Srichandan et al. 2019). In most cases, consortia of microorganisms and biofilm formation have an important role in improving and enhancing biomining, compared to single-species approaches. Moreover, biotechnological and synthetic biology techniques can be applied to ameliorate microbial resistance to toxic compounds and enhance metal extraction. All these concepts can be applied to space biomining.

#### Chemolithotroph microorganisms

This first group includes the most commonly used and characterized biomining organisms, autotrophic iron-oxidising and/or sulphur-oxidising microorganisms which thrive at low pH. Low pH allows many metal ions, such as iron, to remain in solution, although neutral-pH biomining is possible (Blowes et al. 2003). These microorganisms include mesophilic bacteria (optimum 28–37 °C), moderate thermophilic bacteria (40–60 °C) and thermophile chemolithotrophs (60–80 °C), which commonly belong to archaea (Bosecker 1997; Asghari et al. 2013; Srichandan et al. 2019). The genera *Acidithiobacillus, Thiobacillus* and *Leptospirillium* contain a large proportion of these microorganisms, and they have been often isolated from mine-drainage sites (Blowes et al. 2003). Some archaea include the genera *Acidianus*, *Ferroplasma*, *Metallosphaera* and *Sulfolobus* (Schippers et al. 2013). *Acidithiobacillus ferrooxidans*, an iron and sulphur oxidizing microorganism, and *Acidithiobacillus thiooxidans*, a strictly sulphur oxidizing bacterium, are two of the most commonly used bacteria in bioleaching (Levicán et al. 2008).

On Earth, metals of interest are often embedded into sulphidic minerals (e.g., iron in pyrite FeS_2_, copper in chalcopyrite CuFeS_2_, and molybdenum in molybdenite MoS_2_). The processes of metal dissolution performed by chemolithotrophs includes the biological oxidation of Fe^2+^ to Fe^3+^, which in turn oxidises the sulphide mineral to sulphate and sulphuric acid. This latter maintains the low pH required to improve metal solubilisation (Olson et al. 2003; Asghari et al. 2013; Schippers et al. 2013; Singh and Cameotra 2015; Marrero et al. 2020). Metal sulphide dissolution can occur by two main pathways, depending on the mineral species (i.e., electronic configuration and consequent reactivity to proton attack) and named after the intermediate that is formed: (a) thiosulphate (S_2_O_3_^2−^) pathway, enabling the solubilization of acid-insoluble metal sulphides minerals, and (b) polysulphide (H_2_S_n_) pathway, which can solubilize acid-soluble minerals (Schippers and Sand 1999; Rohwerder et al. 2003; Marrero et al 2020). The latter can be solubilized by sulphur-oxidizing bacteria, since the process does not necessarily require Fe^3+^. These microorganisms are also required to dissolve the elemental sulphur which is formed during the process (Rohwerder et al. 2003). Metal sulphide dissolution can occur by contact and non-contact mechanisms, although it has been recently suggested that the two mechanisms are similar (Sand et al. 2001; Rohwerder et al. 2003; Srichandan et al. 2019). Details on these, and on the importance of biofilms for bioleaching, are discussed in Sect. 3.2.4. More recently, reductive dissolution of ferric ion in oxidized ores (e.g. laterites) from *A. ferrooxidans* has been demonstrated. In this process, anaerobic conditions can be used as Fe^3+^ is the terminal electron acceptor (Hallberg et al. 2011; Johnson et al. 2013; Singh and Cameotra 2015). Elemental sulphur is, however, required as an energy source (Hallberg et al. 2011; Johnson et al. 2013; Singh and Cameotra 2015), and the mechanisms still need to be fully elucidated (Singh and Cameotra 2015; Marrero et al. 2020).

**Fig. 1:**
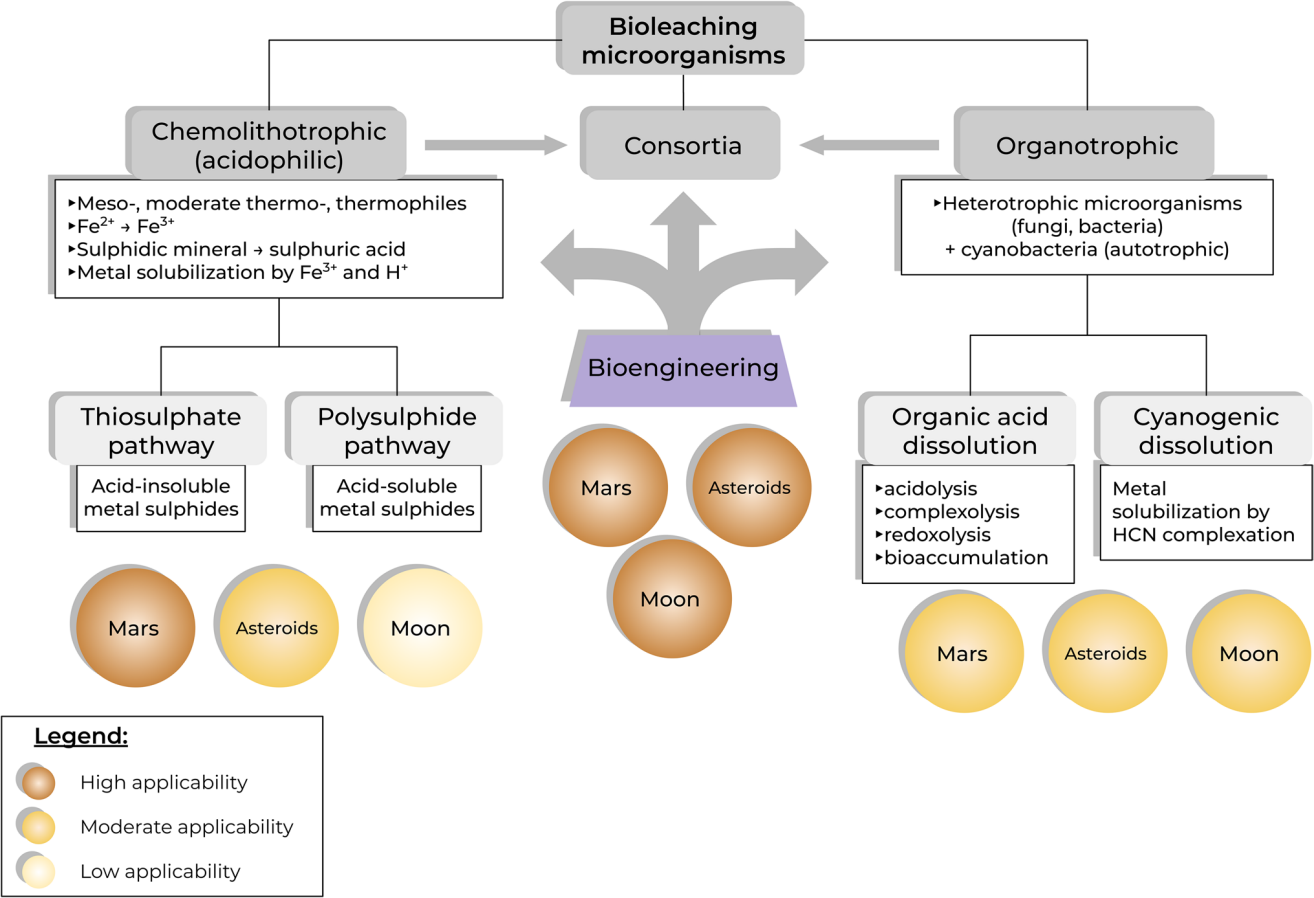
Diagram summarizing the main types of biomining/bioleaching microorganisms and mechanisms, and their potential space applicability taking into account the general surface composition and mineral content of Mars, Moon and asteroids. The central ‘bioengineering’ panel highlights the potential use of bioengineering and synthetic biology approaches to enhance and overcome possible limitations of *wild type* microorganisms. Sphere colour indicates the tentative applicability to planetary bodies, based on the general crust composition. Other parameters (atmosphere, pressure, gravity, temperature, etc.) have not been considered here

The use of chemolithotroph microorganisms in space biomining has two main advantages: first, the mechanisms have been widely characterized and second, they do not require the presence of organic compounds to grow (Averesch 2021). Growth, interaction and metal extraction from space rocks/regolith or analogues using chemolithotroph microorganisms have been demonstrated (González-Toril et al. 2005; Gronstal et al. 2009; Kölbl et al. 2017; Tait et al. 2017; Milojevic et al. 2021). However, as discussed in Sect. 3.1, mineral composition of space rocks of biomining interest can differ widely not only among planetary bodies, but also within the same planet or asteroid. This type of bioleaching microorganisms could not be applied, or be less efficient, to space rocks with low sulphur content, for instance the Moon, some asteroids and potentially some Martian regions (Fig. 1, see also Sect. 3.1).

#### Organotroph microorganisms

The second group of bioleaching microorganisms includes organotrophic bacteria and fungi. These are heterotrophic organisms which require organic compounds as a source of carbon for their metabolism, and they can solubilize oxidic, silicious and carbonaceous materials (Asghari et al. 2013). The metal solubilization of the ore occurs by the action of (i) organic acids or (ii) hydrogen cyanide produced by the bacterial or fungal cell. In this case, bioleaching can occur at circumneutral or slightly alkaline pH (Bosecker 1997). (i) Regarding the first mechanism, fungi have been particularly characterized for their organic acid-mediated bioleaching capacity, which can occur by (a) acidolysis, (b) complexolysis, (c) redoxolysis and (d) bioaccumulation (Asghari et al. 2013; Srichandan et al. 2019). (a) In acidolysis, protons from the organic acid react with the mineral surface, weakening the bonds and consequently releasing the metal of interest. Concurrently, (b) complexolysis can also happen, in which complexation between the metal and the organic acid, or more rarely amminoacids (Maneesuwannarat et al. 2019), occurs. (c) During redoxolysis, metal solubilization is promoted by its enzymatic oxidation or reduction, while in (d) bioaccumulation the soluble metal ions can be transported through the cell membrane and accumulate as solid particle within the cell or in vacuoles (Asghari et al. 2013; Srichandan et al. 2019). The organic acids identified to be associated with bioleaching include citric acid, gluconic acid, oxalic acid, citramalic acid, acetic acid, succinic acid and itaconic acid (Gadd 1999; Adeleke et al. 2010; Amin et al. 2014; Brisson et al. 2016, 2020). Fungal bioleaching species include, but is not limited to, *Penicillium* spp. (Franz et al. 1991; Bosecker 1997; Ambreen et al. 2002; Adeleke et al. 2010), *Aspergillu*s spp, (Brisson et al. 2016; Din et al. 2020) and *Paecilomyces* spp. (Brisson et al. 2016, 2020). Bacterial species include *Acinetobacter*, *Pseudomonas* and *Bacillus* spp. (Reed et al. 2016; Rozas et al. 2017; Barnett et al. 2018; Giese et al. 2019). Brisson and colleagues (Brisson et al. 2020) recently performed a thorough metabolomic study which suggested that bioleaching of a given element could be mediated by specific organic acids, rather than organic acids in general. (ii) The second group of organotrophic bioleaching microorganisms include species producing hydrogen cyanide (HCN, also called cyanogenic microorganisms), that use glycine for its production. HCN formation is usually an aerobic process (Srichandan et al. 2019). These organisms can perform bioleaching at mild temperatures (25–35 °C) and a circum-neutral to basic pH (7–11; Valix 2017). Cyanide forms complexes with the metals, whose solubility in water and chemical stability are highly increased. Many metal and metalloids, including precious elements such as gold, platinum and silver, have been successfully extracted from electronic waste using cyanogenic microorganisms, which include *Pseudomona*s spp., *Bacillus megaterium*, *Escherichia coli* and *Chromobacterium violaceum* (Valix 2017). Notably, cyanobacteria have been shown to perform bioleaching as well (Cecal et al. 2000; Singh 2020). Despite being autotrophic organisms, their bioleaching mechanisms are similar to those of organotrophic microorganisms and involve biosorption through polysaccharides and organic acids (containing amino, carboxyl, hydroxyl and carbonyl groups) present on the cell surface and in biofilms (Huang et al. 2020; Singh 2020).

Extraction of REEs and vanadium from basalt (Mars and Moon analogue rock) using organotrophic bacteria (*Sphingomonas desiccabilis* and *Bacillus subtilis*) was successfully performed on the ISS under Martian gravity and microgravity (Cockell et al. 2020, 2021), while copper, iron and magnesium were extracted by *Cupriavidus metallidurans* from basalt after three months of spaceflight (Byloos et al. 2017). Although used also for extraction from sulphide minerals (Johnson and Roberto 1997), these microorganisms are particularly useful for recovery of metals from non-sulphidic minerals and industrial waste that do not contain the sulphur necessary to sustain chemolithotroph growth (Asghari et al. 2013; Hosseini Nasab et al. 2020). Hence, organotrophic microorganisms with demonstrated biomining capacity could be applied to space biomining to extract elements from those rocks and regolith with low sulphur content, for instance lunar rocks (see also Sect. 3.1). A tentative diagram showing potential applications of biomining mechanisms to space, based on general surface composition of planetary bodies, is shown in Fig. 1. The diagram is not intended as exhaustive or definitive, as other parameters have not been considered, but it shows that careful selection of biomining mechanisms and microorganisms is needed when planning for space applications, as indeed for any space bioengineering and biomanufacturing process (Averesch 2021).

#### Biomining consortia

Consortia of microorganisms, rather than single species, are often used in biomining, as they can perform different reactions which eventually lead to the metal extraction (Rawlings and Johnson 2007). In many cases the consortium has increased bioleaching efficacy. Qiu et al. (2015) showed how bioleaching of copper from chalcopyrite was enhanced in a co-culture of *A. ferrooxidans* and *A. thiooxidans* compared to single cultures (Qiu et al. 2005). The roles that microorganisms can perform in a consortium include the oxidation of the ferrous fraction, the generation of a low pH by oxidation of the sulphur moiety and consequent acidification of the solutions, and the degradation of the organic compounds formed in the process (Johnson 2018). Although some bioleaching microorganisms can perform multiple roles (e.g., *A. ferrooxidans*), others can perform a single role in the process, such as *A. thiooxidans*, being a strict sulphur-oxidizing bacterium (Blowes et al. 2003; Levicán et al. 2008). Heterotrophic consortia and mixes of heterotrophic–autotrophic consortia have also been studied (Bryan et al. 2015; Valix 2017). Selection of the appropriate consortium for a given biomining application can occur by a “top-down” approach, in which a mix of microorganisms is naturally selected by growing in the presence of a certain mineral, or by a “bottom-up” approach, in which a consortium is designed by selecting required parameters (Rawlings and Johnson 2007). Bioengineering of consortia has been also attempted (Rawlings and Johnson 2007; Brune and Bayer 2012; Gumulya et al. 2018). All these approaches are promising from a space biomining perspective, although they may be limited by the necessity to optimize consortia using rock and regolith simulant, when real space samples are not available.

The possibility to use heterotrophic organisms for non-sulphidic mineral dissolution in space biomining has been discussed. While using these microorganisms may present many other advantages (Averesch 2021), one disadvantage is certainly their requirement for a carbon source. This could be overcome by using a consortium containing heterotrophic biomining microorganisms and cyanobacteria: the latter could produce the organic compounds, and the oxygen, necessary to sustain growth and organic acid production (Verseux et al. 2016; Billi et al. 2021) necessary for biomining in space, without the need to provide these nutrients from external sources. The oxygen production would be useful, as it could sustain aerobic biomining in general. Moreover, cyanobacteria (particularly *Anabaena cylindrica*) have been demonstrated to successfully leach mineral elements from basalt and anorthosite (Olsson-Francis and Cockell 2010). An attempt to test the efficacy of bioleaching of asteroidal material performed by a small artificial heterotrophic consortium (the bacterium *S. desiccabilis* plus the fungus *P. simplicissimum*) in microgravity has been completed on the ISS (BioAsteroid, data in preparation).

#### The role of biofilm formation

Microbially mediated leaching of sulphidic minerals can occur by contact and non-contact interaction, although it has been recently suggested that the two mechanisms overlap. In the case of direct interaction, the microbial cell is in contact with the mineral surface through the extracellular polymeric substances (EPS), and the sulphur fraction is oxidised to sulphate by the Fe^3+^-glucuronic acids complex in the EPS. In the non-contact mechanism, instead, the microbial planktonic cell acts by oxidizing the Fe^2+^ and reduced sulphur species that are dissolved in the solution (Sand et al. 2001; Blowes et al. 2003). Biofilm formation is important for both interactions, as it not only enhances the contact of microbial cells with the rock surface, but also increases the concentration of ions and compounds that allow bioleaching, the communication between cells of same or different species, and the resistance to toxic metals and compounds (Noël et al. 2010; Bellenberg et al. 2014; Yu et al. 2014; Gumulya et al. 2018). Indeed, biofilm enhances organotrophic bioleaching as well, through the action of the compounds embedded in the matrix (EPS), including polysaccharides and organic acids (Rohwerder et al. 2003; Yu et al. 2014; Gumulya et al. 2018; Huang et al. 2020). When biofilms may limit the access to the substrate, microorganisms have been demonstrated to modify EPS accordingly (Rohwerder et al. 2003).

Many studies on biofilm formation in space demonstrated the production of thicker or structural different biofilms during spaceflights (McLean et al. 2001; Zea et al. 2018, 2020). A study conducted on board the ISS interrogated biofilm formation and found that, under certain conditions, a novel “column and canopy” structure was observed in microgravity (Kim et al. 2013). The BioRock experiment studied microbe–mineral interaction and bioleaching from basalt rock of three heterotrophic bacterial species, showing a positive biofilm formation for *S. desiccabilis* and *C. metallidurans* within the rock surface under all the gravity condition tested (microgravity, Martian and terrestrial gravities), particularly around edges and cavities (Santomartino et al. in preparation). *S. desiccabilis* and *B. subtilis* were able to extract useful elements under different gravity regimens during the same experiment, suggesting an uninfluential role of gravity on microbial bioleaching mechanisms (Cockell et al. 2020, 2021). The data on biofilm formation and growth will, therefore, provide useful insights into the effects of biofilm formation in space biomining.

#### Bioengineering

In the presence of mine ore or waste, microbial growth can be hindered by toxic compounds, heavy metals and stressor factors. Therefore, it comes as no surprise that many extremotolerant and extremophilic microorganisms have been isolated as bioleaching agents. This characteristic of bioleaching organisms is, by itself, of great use when considering space biomining, since extraterrestrial regolith can often bear toxic compounds, such as perchlorates (Hecht et al. 2009; Wadsworth and Cockell 2017). When not endogenous, resistance could be developed by adaptive evolution, random mutagenesis or bioengineering and synthetic biology approaches (Gumulya et al. 2018). Engineering of single species or a microbial consortium to enhance desired characteristics, or reduce the impact of the undesired ones, is possible, although the success rate of the engineering approach depend on the recalcitration of some microorganisms to genetic manipulation (Rawlings and Johnson 2007; Brune and Bayer 2012; Johnson et al. 2013). Synthetic biology approaches can be used to improve the tolerance, and therefore the output, of biomining microorganisms. The concept is not trivial, as bioengineering has been optimized for a few microbial species (e.g., *E. coli* strains), mainly heterotrophic (Averesch 2021), while tools are rarely available for environmental and undomesticated samples. Gumulya et al. (2018) provides a list of tools developed specifically for a few biomining microorganisms, including *A. ferrooxidans* and *A. thiooxidans*. In many cases, synthetic biology has been used to improve the resistance of *E. coli* strains to acid, thermal or heavy metal stress, to mention a few. This is achieved by heterologous expression of proteins of interest, often isolated from bioleaching microorganisms (Gumulya et al. 2018). Nevertheless, attempts to engineer specific biomining microorganisms (Gumulya et al. 2018) and biofilms for biomining applications (Buetti-Dinh et al. 2020) have been performed as well. *Deinococcus radiodurans*, also known for its astrobiological relevant properties such as resistance to polyextremes and space conditions, has been engineered to enhance its bioremediation capacity (Daly 2000). Attempts to genetically enhance acidophilic biomining microorganisms have been reported (reviewed in Gumulya et al. 2018), for instance by expressing arsenic resistance and rusticyanin proteins in *A. ferrooxidans* (Liu et al. 2011, 2013).

Although synthetic biology applications to biomining are still young, approaches to ameliorate resistance to space conditions, to enhance extraction of elements under these, or overcome issues, could be an excellent opportunity for space biomining, and a wide variety of possible applications have been reported (Cockell 2011; Montague et al. 2012; Menezes et al. 2015b; Rothschild 2016; Verseux et al. 2016). Notably, the importance of synthetic biology approaches for ISRU has been proposed (Cockell 2011; Menezes et al. 2015b; Rothschild 2016; Nangle et al. 2020; Averesch 2021).

## Space biomining applications

### Biomining in the context of ISRU and self-sustaining extraterrestrial settlements

One of the most relevant applications for space biomining is in ISRU. In particular, Bio-ISRU refers to the use of biotechnologies to achieve the exploitation of resources from planetary bodies (Brown et al. 2008). However, myriad resources will need to be acquired and processed in situ to enable self-sustaining settlements in space, and many commodities will likely require a combination of physico-chemical and biological approaches (Keller et al. 2021). Transportation to and from the settlement and Earth, and between settlements, will require fuel for the transport vehicles. While methane production for Martian architecture (Ash et al. 1978; Musk 2017) may be partly done via biological methods (Klas et al. 2015), oxygen and hydrogen extraction from lunar regolith for chemically based propulsion (Jamanca-Lino 2021) will likely be performed via physico-chemical processes (Schwandt et al. 2012). Other essential resources arise from human needs: water, oxygen, atmospheric pressure, food and waste management. Water can be found in permanently shadowed craters on the Moon and in different locations on Mars and asteroids; this water can also serve to meet oxygen needs. Oxygen and an inert gas like N_2_ will be needed to reach required atmospheric pressures (Niederwieser et al. 2019).

In this context, biomining could be one of the methods to provide the structural metals (e.g., iron, nickel, vanadium, copper) for construction of buildings and machinery, or to build electronic devices and magnets (e.g., REEs, silicon; Cockell 2010, 2011; Montague et al. 2012; Menezes et al. 2015b; Rothschild 2016; Verseux et al. 2016). Bioleaching microorganisms could also be applied to recycle useful elements from electric waste (Brandl et al. 1999; Rozas et al. 2017; Srichandan et al. 2019).

**Fig. 2:**
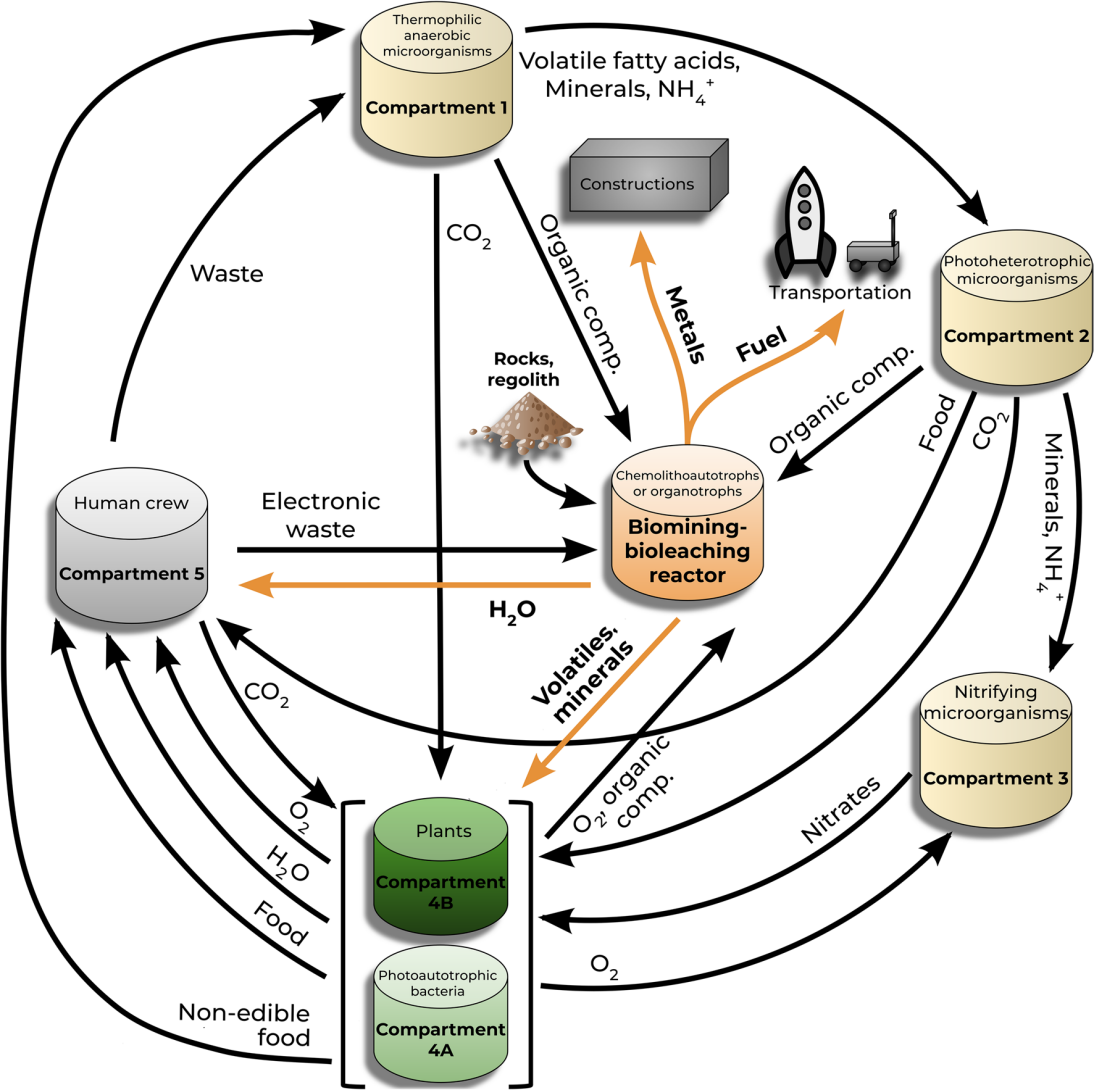
Conceptual figure of a biomining/bioleaching compartment in the context of BLSS, based on the regenerative life-support systems MELiSSA project design (Gòdia et al. 2002; Lasseur and Mergeay 2021). Potential useful elements produced by this compartment are shown in orange

Apart from structural materials, bioleaching of regolith and rocks could provide water, useful volatiles (e.g., oxygen from the dissolution of oxide minerals, hydrogen, carbon, sulphur) and mineral nutrients (e.g., potassium, sodium, calcium, phosphorus, sulphur) essential for life (Cockell 2010, 2011; Montague et al. 2012; Menezes et al. 2015b; Rothschild 2016; Verseux et al. 2016; Huang et al. 2020). Biomining is not intrinsically a traditional part of BLSS (Eckart 1992). However, the principal and secondary (e.g., waste) products from bioleaching discussed so far could be used to sustain biological compartments in life-support systems, including human crew (see for instance the ESA’s MELiSSA life support project; Gòdia et al. 2002; Lasseur and Mergeay 2021). In turn, bioleaching microorganisms (particularly aerobic and heterotrophic ones) could benefit from waste materials produced from the regenerative support system (Billi et al. 2021; Keller et al. 2021). Considering all this, the addition of a biomining/bioleaching compartment could be a beneficial addition to supply basic chemical requirements for a BLSS, and vice versa (Fig. 2).

### Bioremediation

Similar chemical reactions to those involved in biomining are used to perform bioremediation on Earth, to eliminate toxic and polluting compounds from soil and waters (Gadd 2010). Extremophiles and extremotolerant microorganisms (Jeong and Choi 2020), fungi (e.g., *Aspergillus* and *Penicillium* spp.; Yamada-Onodera et al. 2002; Din et al. 2020) and other bacterial species (for instance *Sphingomonas*; Chen 2012) are often used. The capacity to remove heavy metals, radioactive species (uranium), acids (including acid mine drainage produced through mining and biomining processes), organic pollutants and salts has been demonstrated (Gadd 2010; Brune and Bayer 2012). It is worth mentioning that many of these species can indeed thrive in (extremophiles and polyextremophiles) or tolerate (extremotolerant and polyextremotolerant) a variety of extreme conditions, which might come in handy in space. To mention a few examples, many chemolithotrophic microorganisms used in biomining, such as *Acidithiobacillus* and *Ferroplasma,* are acidophiles, while *Sulfolobus* and *Metallosphae*ra are both acidophiles and thermophiles (Rohwerder et al. 2003; Coker 2016). Regarding organotrophic microorganisms, *A. niger* spores demonstrated resistance to high doses of radiations (Cortesão et al. 2020), while *S. desiccabilis* biofilms could withstand desiccation and Martian brines (Stevens et al. 2019), for instance.

The lunar and Martian surfaces contain a number of toxic compounds that could hinder not only biomining, but also the settlement itself. Examples include perchlorates on Mars (Hecht et al. 2009; Kounaves et al. 2014) and toxic lunar dusts (Linnarsson et al. 2012). Microorganisms performing biomining could also be selected for bioremediation approaches to ameliorate these components by making them less toxic or removing them. A recent study identified the genes involved in perchlorate resistance from microorganisms isolated from the Atacama desert (Díaz-Rullo et al. 2021). A *Chroococcidiopsis* species and its derivate (CCMEE 029 and CCMEE 029 P-MRS, respectively) had no negative effect to perchlorate exposure, and the biomass produced could be used to feed an heterotrophic bacterial species (*E. coli*; Billi et al. 2021). A new record for perchlorate tolerance was reported for the fungus *Planococcus halocryophilus* (Heinz et al. 2020). *Shewanella onediensis* was found slightly impacted by perchlorates (Volger et al. 2020), and it was able to reduce chromium, although growth was inhibited at high concentrations of this metal (Middleton et al. 2003). Moreover, biofilms could be used to settle dangerous dusts and enrich regolith with nutritious elements, which will allow the fertilization of soils (Liu et al. 2008; Cockell 2011; Mergelov et al. 2018; Eichler et al. 2021).

### Asteroid biomining

The concept of asteroid mining is slightly different and deserves a separate discussion. While biomining on Mars and the Moon will likely have the aim to directly sustain extraterrestrial settlements located on these planetary bodies, there is no current interest in creating a stable human presence on asteroids (although in the long term this could change). This poses the problem of transporting the extracted materials to their final destination, hence the viability of the process depends on the costs, which depend on the richness of elements and their location (Gertsch 1992), and of course on the engineering constraints. Asteroids of interest from the biomining perspective belong to either the near-Earth objects or the asteroid belt, beyond Mars orbit. For lunar settlements or return to Earth, near-Earth objects could be more appropriate, while for Martian settlements objects from asteroid belt could be used (Gaffey 1992; Gertsch 1992).

Molecules of interest from the asteroid biomining perspective are water, volatiles and carbon compounds, expensive (PGE, gold, silver) and common metals (Klas et al. 2015). We discussed in Sect. 3.1.v the wide variety of asteroids and their composition, as well as the fact that our knowledge on these is limited by data coming from the available meteorites and the few returning samples from space missions (Metzger and Britt 2020). It has been estimated that the total number of minor asteroids in the asteroid belt is highly underestimated (Krasinsky et al. 2002). These considerations imply that a wider knowledge on asteroid type and composition is necessary to select objects of interest for space biomining.

Asteroid biomining is gaining increasing interest, and a variety of companies are forming to develop engineering strategies (Lewicki et al. 2013), and scientists are assessing microbial growth and colonization of meteorites (Klas et al. 2015; Tait et al. 2017; Milojevic et al. 2019), including the abovementioned BioAsteroid experiment, showing bacterial and fungal mediated leaching of an L-chondrite on the ISS (data under analysis).

### Benefits for Earth

Scientific research topics are naturally interconnected. Owing to the harshness of the space environment, space exploration requires the most advanced human ingenuity and scientific technologies. Once these are developed, they would be available for terrestrial applications and benefit the quality of life on Earth (ISECG 2013). Space microbiology also has a significant impact in our everyday life (Horneck et al. 2010). With this view in mind, it is expected that advances in space biomining will eventually benefit terrestrial biomining technologies (Metzger 2016). It has been proposed that biomining of specific planetary bodies, for instance some asteroids and even the Moon (Carter 1992), could provide useful elements for terrestrial use. However, at present this has not been found to be a viable opportunity. Nevertheless, many realistic benefits can be listed.

As mentioned before, the BioRock experiment demonstrated the ability of *S. desiccabilis* to extract REEs and vanadium under a variety of gravity conditions on the ISS (Cockell et al. 2020, 2021). This was the first demonstration of the ability of *S. desiccabilis* to perform biomining, providing a novel microorganism for terrestrial applications. Improvement of biomining technologies, including amelioration of strains for the extraction of elements from low-grade ores and under extreme conditions could provide tools for similar aims on Earth. Designing of highly efficient biomining reactors and hardware would be another applicable benefit. The same concept could be easily applied to bioremediation, hence to environmental issues as follows: microorganisms with demonstrated tolerance and capacity to bioaccumulate and remediate toxic compounds under the harsh Martian and lunar conditions could be used for terrestrial purposes, or inform us on how to achieve similar goals here.

Augmentation of sustainable approaches by learning from space biotechnologies will be a great benefit for Earth. The major space agencies aligned with the United Nations Sustainable Development Goals (https://sdgs.un.org/goals), and these principles can be applied to human space exploration. Biomining is widely recognized to be more environmental friendly in respect to traditional mining approaches (Jerez 2017a); hence learning how to improve bioleaching from advancements in the challenging field of space biomining would also have a positive environmental impact on Earth. Moreover, improving the design of self-sustaining extraterrestrial settlements, for instance by including a biomining compartment, electronic waste recycling and synthetic biology approaches to engineer biomining microorganisms to use different waste as nutrients (e.g., plastics), would inform on terrestrial strategies to improve circular economy approaches.

## Conclusions

As the goal for space exploration expands toward establishing permanent settlements in space, space biomining is generating an increasing interest. The main space agencies have recognized its potential in their roadmaps. Many are the advantages, as terrestrial biomining is considered a sustainable approach in both the economic and environmental terms (Jerez 2017a). Moreover, many rocks in the Solar System would be considered low-grade ores for terrestrial standards (Cockell and Santomartino, in press), and biomining on Earth is commonly used to process mine waste, dumps and tailings (Bosecker 1997; Mishra and Rhee 2014). The use of bioleaching microorganisms could also be applied to bioremediation, recycling of waste and to support BLSS.

Although promising, the science around space biomining is still relatively young. Considering the differences between terrestrial and extraterrestrial conditions, including rock type and ore availability, a direct application of established terrestrial biomining techniques may not be always possible. Energetic and metabolic demands need to be taken into account. Engineering requirements for space biomining reactors will require temperature controls, aeration and stirring under complex and harsh conditions. While most terrestrial biomining knowledge is based on autotrophic microorganisms able to break down sulphidic minerals without the need of carbon compounds, many rocks in the Solar Systems may present a low sulphur content. In these cases, heterotrophic microorganisms could be a better choice, but they would require organic compounds. Moreover, most bioleaching reactions are aerobic, and oxygen itself will be a commodity in space. A careful selection of the most appropriate techniques and microorganisms will be required for any given application, and the optimal solution could include complementary systems (Averesch 2021). Ethic and planetary protection discussions are outside of the aim of this review. Nevertheless, it is necessary to take them into account when planning the preferred method for mining or ISRU in a specific region or planetary body, as they could pose a further limitation.

Biomining is one of the possible approaches to achieve ISRU, and its selection over other methods should at least consider all the parameters discussed here. Nevertheless, its advantages make it pivotal to invest in terrestrial and space-based research of specific methods for space applications. This includes learning more on the effects of space conditions on biomining and bioremediation, expanding our knowledge on organotrophic, cyanobacteria and community-based bioleaching mechanisms, on anaerobic biomining, and investigating the use of synthetic biology to overcome limitations.

## Abbreviations

BLSS: Biological life support system
EPS: Extracellular polymeric substances
GCR: Galactic cosmic rays
ISRU: In-situ resource utilization
ISS: International Space Station
PGE: Platinum group elements
REEs: Rare earth elements
SEP: Solar energetic particles

## References

[CR1] Adeleke R, Cloete E, Khasa D (2010). Isolation and identification of iron ore-solubilising fungus. S Afr J Sci.

[CR2] Ambreen N, Bhatti HN, Bhatti TM (2002). Bioleaching of bauxite by *Penicillium simplicissimum*. J Biol Sci.

[CR3] Amin MM, Elaassy IE, El-Feky MG, Sallam ASM, Talaat MS, Kawady NA (2014). Effect of mineral constituents in the bioleaching of uranium from uraniferous sedimentary rock samples, Southwestern Sinai, Egypt. J Environ Radioact.

[CR4] Anand M, Crawford IA, Balat-Pichelin M, Abanades S, Van Westrenen W, Péraudeau G (2012). A brief review of chemical and mineralogical resources on the Moon and likely initial in situ resource utilization (ISRU) applications. Planet Space Sci.

[CR5] Arai T, Takeda H, Yamaguchi A, Ohtake M (2008). A new model of lunar crust: asymmetry in crustal composition and evolution. Earth Planets Sp.

[CR6] Asghari I, Mousavi SM, Amiri F, Tavassoli S (2013). Bioleaching of spent refinery catalysts: a review. J Ind Eng Chem.

[CR7] Ash RL, Dowler WL, Varsi G (1978). Feasibility of rocket propellant production on Mars. Acta Astronaut.

[CR8] Averesch NJH (2021). Choice of microbial system for in-situ resource utilization on mars. Front Astron Sp Sci.

[CR9] Barnett MJ, Palumbo-Roe B, Gregory SP (2018). Comparison of heterotrophic bioleaching and ammonium sulfate ion exchange leaching of rare earth elements from a Madagascan ion-adsorption clay. Minerals.

[CR10] Baumstark-Khan C, Facius R (2002). Life under conditions of ionizing radiation. Astrobiology—the quest for the conditions of life.

[CR11] Bellenberg S, Díaz M, Noël N, Sand W, Poetsch A, Guiliani N (2014). Biofilm formation, communication and interactions of leaching bacteria during colonization of pyrite and sulfur surfaces. Res Microbiol.

[CR12] Berliner AJ, Hilzinger JM, Abel AJ, McNulty MJ, Makrygiorgos G, Averesch NJH (2021). Towards a biomanufactory on mars. Front Astron Sp Sci.

[CR13] Billi D, Mosca C, Fagliarone C, Napoli A, Verseux C, Baqué M (2019). Exposure to low Earth orbit of an extreme-tolerant cyanobacterium as a contribution to lunar astrobiology activities. Int J Astrobiol.

[CR14] Billi D, Gallego Fernandez B, Fagliarone C, Chiavarini S, Rothschild LJ (2021). Exploiting a perchlorate-tolerant desert cyanobacterium to support bacterial growth for in situ resource utilization on Mars. Int J Astrobiol.

[CR15] Blowes DW, Ptacek CJ, Jambor JL, Weisener CG (2003) The geochemistry of acid mine drainage. In: Treatise on geochemistry, pp 149–204. doi:10.1016/B0-08-043751-6/09137-4.

[CR16] Bosecker K (1997). Bioleaching: metal solubilization by microorganisms. FEMS Microbiol Rev.

[CR17] Brandl H, Bosshard R, Wegmann M (1999). Computer-munching microbes: metal leaching from electronic scrap by bacteria and fungi. Process Metall.

[CR18] Brisson VL, Zhuang WQ, Alvarez-Cohen L (2016). Bioleaching of rare earth elements from monazite sand. Biotechnol Bioeng.

[CR19] Brisson VL, Zhuang WQ, Alvarez-Cohen L (2020). Metabolomic analysis reveals contributions of citric and citramalic acids to rare earth bioleaching by a *Paecilomyces* fungus. Front Microbiol.

[CR20] Brounce M, Boyce J, McCubbin FM, Humphreys J, Reppart J, Stolper E (2019). The oxidation state of sulfur in lunar apatite. Am Mineral.

[CR21] Brounce M, Boyce JW, Barnes J, McCubbin FM (2020). Sulfur in the apollo lunar basalts and implications for future sample-return missions. Elements.

[CR22] Brown II, Garrison DH, Jones JA, Allen CC, Sanders G, Sarkisova SA et al (2008) The development and perspectives of Bio-ISRU. In: Joint Annual Meeting of LEAG-ICEUM-SRR.

[CR23] Brune KD, Bayer TS (2012). Engineering microbial consortia to enhance biomining and bioremediation. Front Microbiol.

[CR24] Bryan CG, Watkin EL, McCredden TJ, Wong ZR, Harrison STL, Kaksonen AH (2015). The use of pyrite as a source of lixiviant in the bioleaching of electronic waste. Hydrometallurgy.

[CR25] Buetti-Dinh A, Herold M, Christel S, Hajjami ME, Bellenberg S, Ilie O (2020). Systems biology of acidophile biofilms for efficient metal extraction. Sci Data.

[CR26] Byloos B, Coninx I, Van Hoey O, Cockell C, Nicholson N, Ilyin V (2017). The impact of space flight on survival and interaction of *Cupriavidus metallidurans* CH34 with basalt, a volcanic moon analog rock. Front Microbiol.

[CR27] Campins H, Hargrove K, Pinilla-Alonso N, Howell ES, Kelley MS, Licandro J (2010). Water ice and organics on the surface of the asteroid 24 Themis. Nature.

[CR28] Carter JL (1992) Lunar material resources: an overview. In: Space resources: materials 19–49.

[CR29] Castelein SM, Aarts TF, Schleppi J, Hendrikx R, Böttger AJ, Benz D (2021). Iron can be microbially extracted from Lunar and Martian regolith simulants and 3D printed into tough structural materials. PLoS ONE.

[CR30] Cecal A, Humelnicu D, Popa K, Rudic V, Gulea A, Palamaru I (2000). Bioleaching of UO22+ ions from poor uranium ores by means of cyanobacteria. J Radioanal Nucl Chem.

[CR31] Chen W (2012). The study of bioremediation on heavy metal of cultured seawater by *Sphingomonas* sp. XJ2 immobilized *Sphingomonas* strain. Adv Mater Res.

[CR32] Clark BC (1993). Geochemical components in Martian soil. Geochim Cosmochim Acta.

[CR33] Cockell CS (2010). Geomicrobiology beyond Earth: Microbe-mineral interactions in space exploration and settlement. Trends Microbiol.

[CR34] Cockell CS (2011). Synthetic geomicrobiology: engineering microbe–mineral interactions for space exploration and settlement. Int J Astrobiol.

[CR35] Cockell CS (2021). Bridging the gap between microbial limits and extremes in space: space microbial biotechnology in the next 15 years. Microb Biotechnol.

[CR36] Cockell CS, Santomartino R, Hessel V, Stoudemire J, Miyamoto H, Fisk ID (2021). Mining and microbiology for the solar system silicate and basalt economy. Space manufacturing resources: earth and planetary exploration applications.

[CR37] Cockell CS, Schuerger AC, Billi D, Friedmann EI, Panitz C (2005). Effects of a simulated martian UV flux on the *Cyanobacterium*, *Chroococcidiopsis sp*. 029. Astrobiology.

[CR38] Cockell CS, Santomartino R, Finster K, Waajen AC, Eades LJ, Moeller R (2020). Space station biomining experiment demonstrates rare earth element extraction in microgravity and Mars gravity. Nat Commun.

[CR39] Cockell CS, Santomartino R, Finster KW, Waajen AC, Loudon C-M, Eades LJ (2021). Microbially-enhanced vanadium mining and bioremediation under micro- and mars gravity on the international space station. Front Microbiol.

[CR40] Coker JA (2016). Extremophiles and biotechnology: Current uses and prospects. F1000Research.

[CR41] Coradini A, Capaccioni F, Erard S, Arnold G, De Sanctis MC, Filacchione G (2011). The surface composition and temperature of asteroid 21 Lutetia as observed by Rosetta/VIRTIS. Science.

[CR42] Cortesão M, de Haas A, Unterbusch R, Fujimori A, Schütze T, Meyer V (2020). *Aspergillus niger* spores are highly resistant to space radiation. Front Microbiol.

[CR43] Daly MJ (2000). Engineering radiation-resistant bacteria for environmental biotechnology. Curr Opin Biotechnol.

[CR44] De Sanctis MC, Ammannito E, McSween HY, Raponi A, Marchi S, Capaccioni F (2017). Localized aliphatic organic material on the surface of Ceres. Science.

[CR45] Díaz-Rullo J, Rodríguez-Valdecantos G, Torres-Rojas F, Cid L, Vargas IT, González B (2021). Mining for perchlorate resistance genes in microorganisms from sediments of a hypersaline pond in Atacama Desert Chile. Front Microbiol.

[CR46] Din G, Hassan A, Rafiq M, Hasan F, Badshah M, Khan S (2020). Characterization of Organic acid producing *Aspergillus tubingensis* FMS1 and its role in metals leaching from soil. Geomicrobiol J.

[CR47] Eckart P (1992) Bioregenerative life support concepts. In: Spaceflight life support and biospherics, p 249–364.

[CR48] Ehlmann BL, Edwards CS (2014). Mineralogy of the Martian surface. Annu Rev Earth Planet Sci.

[CR49] Eichler A, Hadland N, Pickett D, Masaitis D, Handy D, Perez A (2021). Challenging the agricultural viability of Martian regolith simulants. Icarus.

[CR50] El Fadli KI, Cerveny RS, Burt CC, Eden P, Parker D, Brunet M (2013). World meteorological organization assessment of the purported world record 58 °C temperature extreme at el Azizia, Libya (13 September 1922). Bull Am Meteorol Soc.

[CR51] Fahrion J, Mastroleo F, Dussap CG, Leys N (2021). Use of photobioreactors in regenerative life support systems for human space exploration. Front Microbiol.

[CR52] Faraji F, Golmohammadzadeh R, Rashchi F, Alimardani N (2018). Fungal bioleaching of WPCBs using *Aspergillus niger*: observation, optimization and kinetics. J Environ Manag.

[CR53] Franz A, Burgstaller W, Schinner F (1991). Leaching with *Penicillium simplicissimum*: influence of metals and buffers on proton extrusion and citric acid production. Appl Environ Microbiol.

[CR54] Franz HB, Trainer MG, Malespin CA, Mahaffy PR, Atreya SK, Becker RH (2017). Initial SAM calibration gas experiments on Mars: Quadrupole mass spectrometer results and implications. Planet Space Sci.

[CR55] Franz HB, King PL, Gaillard F (2019) Sulfur on Mars from the atmosphere to the core. In: Volatiles in the martian crust ed. E. Inc., pp 119–183. doi:10.1016/B978-0-12-804191-8.00006-4.

[CR56] Gadd GM (1999). Fungal production of citric and oxalic acid: Importance in metal speciation, physiology and biogeochemical processes. Elsevier Masson SAS.

[CR57] Gadd GM (2010). Metals, minerals and microbes: geomicrobiology and bioremediation. Microbiology.

[CR58] Gaffey MJ (1992) Ground-based observation of near-earth asteroids. In: Space resources: materials 50–58

[CR59] Gertsch RE (1992) Asteroid mining. In: Space resources: materials 111–120

[CR60] Giese EC, Carpen HL, Bertolino LC, Schneider CL (2019). Characterization and bioleaching of nickel laterite ore using *Bacillus subtilis* strain. Biotechnol Prog.

[CR61] Gnida A (2020). What do we know about the influence of vacuum on bacterial biocenosis used in environmental biotechnologies ?. Appl Microbiol Biotechnol.

[CR62] Gòdia F, Albiol J, Montesinos JL, Pérez J, Creus N, Cabello F (2002). MELISSA: a loop of interconnected bioreactors to develop life support in Space. J Biotechnol.

[CR63] González-Toril E, Martínez-Frías J, Gómez Gómez JM, Rull F, Amils R (2005). Iron meteorites can support the growth of acidophilic chemolithoautotrophic microorganisms. Astrobiology.

[CR64] Greenwood JP, Mojzsis SJ, Coath CD (2000). Sulfur isotopic compositions of individual sulfides in Martian meteorites ALH840001 and Nakhla: implications for crust-regolith exchanges on Mars. Earth Planet Sci Lett.

[CR65] Gronstal A, Pearson V, Kappler A, Dooris C, Anand M, Poitrasson F (2009). Laboratory experiments on the weathering of iron meteorites and carbonaceous chondrites by iron-oxidizing bacteria. Meteorit Planet Sci.

[CR66] Gumulya Y, Boxall NJ, Khaleque HN, Santala V, Carlson RP, Kaksonen AH (2018). In a quest for engineering acidophiles for biomining applications: challenges and opportunities. Genes (basel).

[CR67] Haberle RM (2015). Solar system/sun, atmospheres, evolution of atmospheres: planetary atmospheres: mars. Second edition.

[CR68] Hallberg KB, Grail BM, Plessis CAD, Johnson DB (2011). Reductive dissolution of ferric iron minerals: a new approach for bio-processing nickel laterites. Miner Eng.

[CR69] Hecht MH, Kounaves SP, Quinn RC, Wesy SJ, Young SMM, Ming DW (2009). Detection of perchlorate and the soluble chemistry of martian soil at the phoenix lander site. Science.

[CR70] Heinz J, Krahn T, Schulze-Makuch D (2020). A new record for microbial perchlorate tolerance: fungal growth in NaClO4 brines and its implications for putative life on Mars. Life.

[CR71] Horneck G, Klaus DM, Mancinelli RL (2010). Space microbiology. Microbiol Mol Biol Rev.

[CR72] Horneck G, Moeller R, Cadet J, Douki T, Mancinelli RL, Nicholson WL (2012). Resistance of bacterial endospores to outer space for planetary protection purposes-experiment PROTECT of the EXPOSE-E Mission. Astrobiology.

[CR73] Hosseini Nasab M, Noaparast M, Abdollahi H, Amoozegar MA (2020). Indirect bioleaching of Co and Ni from iron rich laterite ore, using metabolic carboxylic acids generated by *P. putida*, *P. koreensis*, *P. bilaji* and *A. niger*. Hydrometallurgy.

[CR74] Housen KR, Wilkening LL, Chapman CR, Greenberg R (1979). Asteroidal regoliths. Icarus.

[CR75] Huang B, Li DG, Huang Y, Liu CT (2018). Effects of spaceflight and simulated microgravity on microbial growth and secondary metabolism. Mil Med Res.

[CR76] Huang W, Ertekin E, Wang T, Cruz L, Dailey M, DiRuggiero J (2020). Mechanism of water extraction from gypsum rock by desert colonizing microorganisms. Proc Natl Acad Sci.

[CR77] Hughes DW (1994). The historical unravelling of the diameters of the first four asteroids. Q J R Astron Soc.

[CR78] ISECG (2013) Benefits stemming from space exploration. ESA Publ. 1–22. http://www.globalspaceexploration.org/wordpress/wp-content/uploads/2013/10/Benefits-Stemming-from-Space-Exploration-2013.pdf.

[CR79] Jaafar R, Al-Sulami A, Al-Taee A, Aldoghachi F, Napes S (2015). Biosorption and bioaccumulation of some heavy metals by *Deinococcus Radiodurans* isolated from soil in basra governorate—Iraq. J Biotechnol Biomater.

[CR80] Jamanca-Lino G (2021). Space resources engineering: ilmenite deposits for oxygen production on the moon. Am J Min Metall.

[CR81] Jeong SW, Choi YJ (2020). Extremophilic microorganisms for the treatment of toxic pollutants in the environment. Molecules.

[CR82] Jerez CA (2017). Biomining of metals: how to access and exploit natural resource sustainably. Microb Biotechnol.

[CR83] Jerez CA (2017). Metal extraction and biomining.

[CR84] Johansson KR (1992) Bioprocessing of ores: application to space resources. In: Space resources: materials, pp 222–241

[CR85] Johnson DB (2014). Biomining-biotechnologies for extracting and recovering metals from ores and waste materials. Curr Opin Biotechnol.

[CR86] Johnson DB (2018). The evolution, current status, and future prospects of using biotechnologies in the mineral extraction and metal recovery sectors. Minerals.

[CR87] Johnson DB, Roberto FF (1997). Heterotrophic Acidophiles and their roles in the bioleaching of sulfide minerals. Biomining.

[CR88] Johnson DB, Grail BM, Hallberg KB (2013). A new direction for biomining: extraction of metals by reductive dissolution of oxidized ores. Minerals.

[CR89] Jolliff B (2008). Lunar mineralogy and global distribution on the moon’s surface. EPSC Abstr.

[CR90] Jolliff BL, Gillis JJ, Haskin LA, Korotev RL, Wieczorek MA (2000). Major lunar crustal terranes: Surface expressions and crust-mantle origins. J Geophys Res E Planets.

[CR91] Keller RJ, Porter W, Goli K, Rosenthal R, Butler N, Jones JA (2021). Biologically-based and physiochemical life support and in situ resource utilization for exploration of the solar system—reviewing the current state and defining future development needs. Life.

[CR92] Kim W, Tengra FK, Young Z, Shong J, Marchand N, Chan HK (2013). Spaceflight Promotes Biofilm Formation by *Pseudomonas aeruginosa*. PLoS ONE.

[CR93] Klas M, Tsafnat N, Dennerley J, Beckmann S, Osborne B, Dempster AG (2015). Biomining and methanogenesis for resource extraction from asteroids. Space Policy.

[CR94] Kölbl D, Pignitter M, Somoza V, Schimak MP, Strbak O, Blazevic A (2017). Exploring fingerprints of the extreme thermoacidophile *Metallosphaera sedula* grown on synthetic martian regolith materials as the sole energy sources. Front Microbiol.

[CR95] Kounaves SP, Chaniotakis NA, Chevrier VF, Carrier BL, Folds KE, Hansen VM (2014). Identification of the perchlorate parent salts at the Phoenix Mars landing site and possible implications. Icarus.

[CR96] Krasinsky GA, Pitjeva EV, Vasilyev MV, Yagudina EI (2002). Hidden mass in the asteroid belt. Icarus.

[CR97] Lasseur C, Mergeay M (2021). Current and future ways to closed life support systems: virtual MELiSSA conference, Ghent (B) (3–5/11/2020) a review. Ecol Eng Environ Prot.

[CR98] Lauretta DS, DellaGiustina DN, Bennett CA, Golish DR, Becker KJ, Balram-Knutson SS (2019). The unexpected surface of asteroid (101955) Bennu. Nature.

[CR99] Lawson SL, Feldman WC, Lawrence DJ, Moore KR, Elphic RC, Belian RD (2005). Recent outgassing from the lunar surface: the lunar prospector alpha particle spectrometer. J Geophys Res E Planets.

[CR100] Levicán G, Ugalde JA, Ehrenfeld N, Maass A, Parada P (2008). Comparative genomic analysis of carbon and nitrogen assimilation mechanisms in three indigenous bioleaching bacteria: predictions and validations. BMC Genomics.

[CR101] Lewicki C, Diamandis P, Anderson E, Voorhees C, Mycroft F (2013). Planetary resources—the asteroid mining company. New Sp.

[CR102] Lewis JS (1992). Asteroid resources. In: Space resources: materials, p 59–78

[CR103] Linnarsson D, Carpenter J, Fubini B, Gerde P, Karlsson LL, Loftus DJ (2012). Toxicity of lunar dust. Planet Space Sci.

[CR104] Liu Y, Cockell CS, Wang G, Hu C, Chen L, De Philippis R (2008). Control of lunar and martian dust-experimental insights from artificial and natural cyanobacterial and algal crusts in the desert of Inner Mongolia, China. Astrobiology.

[CR105] Liu W, Lin J, Pang X, Cui S, Mi S, Lin J (2011). Overexpression of rusticyanin in *Acidithiobacillus ferrooxidans* ATCC19859 increased Fe(II) oxidation activity. Curr Microbiol.

[CR106] Liu M, Dong F, Zhang W, Kang W, Nie X, Wei H (2012). Biosorption of uranium by *Deinococcus radiodurans* cells under culture conditions. Adv Mater Res.

[CR107] Liu W, Lin J, Pang X, Mi S, Cui S, Lin J (2013). Increases of ferrous iron oxidation activity and arsenic stressed cell growth by overexpression of Cyc2 in *Acidithiobacillus ferrooxidans* ATCC19859. Biotechnol Appl Biochem.

[CR108] Maneesuwannarat S, Kudpeng K, Yingchutrakul Y, Roytrakul S, vangnai AS, Yamashita M (2019). A possible protein model involved in gallium arsenide leaching by *Cellulosimicrobium funkei*. Miner Eng.

[CR109] Marrero J, Coto O, Schippers A (2020) Metal bioleaching: fundamentals and geobiotechnical application of aerobic and anaerobic acidophiles. In: Biotechnological applications of extremophilic microorganisms (De Gruyter), p 261–288. doi:10.1515/9783110424331-011.

[CR110] Matsumoto T, Harries D, Langenhorst F, Miyake A, Noguchi T (2020). Iron whiskers on asteroid Itokawa indicate sulfide destruction by space weathering. Nat Commun.

[CR111] Mattimore V, Battista JR (1996). Radioresistance of *Deinococcus radiodurans*: Functions necessary to survive ionizing radiation are also necessary to survive prolonged desiccation. J Bacteriol.

[CR112] McLean RJC, Cassanto JM, Barnes MB, Koo JH (2001). Bacterial biofilm formation under microgravity conditions. FEMS Microbiol Lett.

[CR113] McSween HY, Jeffrey Taylor G, Wyatt MB (2009). Elemental composition of the martian crust. Science.

[CR114] Menezes AA, Cumbers J, Hogan JA, Arkin AP (2015). Towards synthetic biological approaches to resource utilization on space missions. J R Soc Interface.

[CR115] Menezes AA, Montague MG, Cumbers J, Hogan JA, Arkin AP (2015). Grand challenges in space synthetic biology. J R Soc Interface.

[CR116] Mergelov N, Mueller CW, Prater I, Shorkunov I, Dolgikh A, Zazovskaya E (2018). Alteration of rocks by endolithic organisms is one of the pathways for the beginning of soils on Earth. Sci Rep.

[CR117] Metzger PT (2016). Space development and space science together, an historic opportunity. Space Policy.

[CR118] Metzger PT, Britt DT (2020). Model for asteroid regolith to guide simulant development. Icarus.

[CR119] Metzger PT, Zacny K, Morrison P (2020). Thermal extraction of volatiles from lunar and asteroid regolith in axisymmetric Crank-Nicolson modeling. J Aerosp Eng.

[CR120] Meyer C (2003) Lunar sample mineralogy. In: NASA Lunar petrographic educational thin section set, 8–9

[CR121] Middleton SS, Latmani RB, Mackey MR, Ellisman MH, Tebo BM, Criddle CS (2003). Cometabolism of Cr(VI) by *Shewanella oneidensis* MR-1 produces cell-associated reduced chromium and inhibits growth. Biotechnol Bioeng.

[CR122] Milojevic T, Kölbl D, Ferrière L, Albu M, Kish A, Flemming RL (2019). Exploring the microbial biotransformation of extraterrestrial material on nanometer scale. Sci Rep.

[CR123] Milojevic T, Albu M, Kölbl D, Kothleitner G, Bruner R, Morgan ML (2021). Chemolithotrophy on the Noachian Martian breccia NWA 7034 via experimental microbial biotransformation. Commun Earth Environ.

[CR124] Minton KW (1996). Repair of ionizing-radiation damage in the radiation resistant bacterium *Deinococcus radiodurans*. Mutat Res DNA Repair.

[CR125] Mishra D, Rhee YH (2014). Microbial leaching of metals from solid industrial wastes. J Microbiol.

[CR126] Moeller R, Raguse M, Reitz G, Okayasu R, Li Z, Klein S (2014). Resistance of *Bacillus subtilis* spore DNA to lethal ionizing radiation damage relies primarily on spore core components and DNA repair, with minor effects of oxygen radical detoxification. Appl Environ Microbiol.

[CR127] Montague M, McArthur GH, Cockell CS, Held J, Marshall W, Sherman LA (2012). The role of synthetic biology for in situ resource utilization (ISRU). Astrobiology.

[CR128] Murphy JR, Leovy CB, Tillman JE (1990). Observations of Martian surface winds at the Viking Lander 1 site. J Geophys Res.

[CR129] Musk E (2017). Making humans a multi-planetary species. New Sp.

[CR130] Nangle SN, Wolfson MY, Hartsough L, Ma NJ, Mason CE, Merighi M (2020). The case for biotech on Mars. Nat Biotechnol.

[CR131] NASA-ASEE (1992) Space resources: materials. NASA Sci Tech Inf Progr 3

[CR132] Nicholson WL, Ricco AJ (2020). Nanosatellites for biology in space: In situ measurement of *Bacillus subtilis* spore germination and growth after 6 months in low earth orbit on the O/OREOS mission. Life.

[CR133] Niederwieser T, Kociolek P, Hoehn A, Klaus D (2019). Effect of altered nitrogen partial pressure on *Chlorellaceae* for spaceflight applications. Algal Res 41

[CR134] Noël N, Florian B, Sand W (2010). AFM & EFM study on attachment of acidophilic leaching organisms. Hydrometallurgy.

[CR135] Ohmura N, Sasaki K, Matsumoto N, Saiki H (2002). Anaerobic respiration Using Fe3+, S0, and H2 in the chemolithoautotrophic bacterium* Acidithiobacillus ferrooxidan*s. J Bacteriolo.

[CR136] Ohtake M, Matsunaga T, Haruyama J, Yokota Y, Morota T, Honda C (2009). The global distribution of pure anorthosite on the Moon. Nature.

[CR137] Okada T, Fukuhara T, Tanaka S, Taguchi M, Arai T, Senshu H (2020). Highly porous nature of a primitive asteroid revealed by thermal imaging. Nature.

[CR138] Olson GJ, Brierley JA, Brierley CL (2003). Bioleaching review part B: progress in bioleaching: Applications of microbial processes by the minerals industries. Appl Microbiol Biotechnol.

[CR139] Olsson-Francis K, Cockell CS (2010). Use of cyanobacteria for in-situ resource use in space applications. Planet Space Sci.

[CR140] Payré V, Fabre C, Sautter V, Cousin A, Mangold N, Deit LL (2019). Copper enrichments in the Kimberley formation in Gale crater, Mars: evidence for a Cu deposit at the source. Icarus.

[CR141] Pollard EC (1965). Theoretical studies on living systems in the absence of mechanical stress. J Theor Biol.

[CR142] Prölss GW (2004) Absorption and dissipation of solar wind energy. In: Physics of the earth’s space environment, pp 349–399. doi:10.1007/978-3-642-97123-5_7.

[CR143] Qiu MQ, Xiong SY, Zhang WM, Wang GX (2005). A comparison of bioleaching of chalcopyrite using pure culture or a mixed culture. Miner Eng.

[CR144] Raafat K, Burnett JA, Chapman T, Cockell CS (2013). The physics of mining in space. A&g.

[CR145] Rawlings DE, Johnson DB (2007). The microbiology of biomining: development and optimization of mineral-oxidizing microbial consortia. Microbiology.

[CR146] Reed DW, Fujita Y, Daubaras DL, Jiao Y, Thompson VS (2016). Bioleaching of rare earth elements from waste phosphors and cracking catalysts. Hydrometallurgy.

[CR147] Rieder R, Economou T, Wänke H, Turkevich A, Crisp J, Brückner J (1997). The chemical composition of martian soil and rocks returned by the mobile alpha proton x-ray spectrometer: preliminary results from the X-ray mode. Science.

[CR148] Righter K, Go BM, Pando KA, Danielson L, Ross DK, Rahman Z (2017). Phase equilibria of a low S and C lunar core: implications for an early lunar dynamo and physical state of the current core. Earth Planet Sci Lett.

[CR149] Rohwerder T, Gehrke T, Kinzler K, Sand W (2003). Bioleaching review part A: progress in bioleaching: fundamentals and mechanisms of bacterial metal sulfide oxidation. Appl Microbiol Biotechnol.

[CR150] Rothschild LJ (2016). Synthetic biology meets bioprinting: enabling technologies for humans on Mars (and Earth). Biochem Soc Trans.

[CR151] Rozas EE, Mendes MA, Nascimento CAO, Espinosa DCR, Oliveira R, Oliveira G (2017). Bioleaching of electronic waste using bacteria isolated from the marine sponge *Hymeniacidon heliophila* (Porifera). J Hazard Mater.

[CR152] Sand W, Gehrke T, Jozsa P, Schippers A (2001). (Bio) chemistry of bacterial leaching—direct vs. indirect bioleaching. Hydro.

[CR153] Santomartino R, Waajen AC, Wit WD, Nicholson N, Parmitano L, Loudon C (2020). No effect of microgravity and simulated mars gravity on final bacterial cell concentrations on the international space station: applications to space bioproduction. Front Microbiol.

[CR154] Schippers A, Sand W (1999). Bacterial leaching of metal sulfides proceeds by two indirect mechanisms via thiosulfate or via polysulfides and sulfur. Appl Environ Microbiol.

[CR155] Schippers A, Hedrich S, Vasters J, Drobe M, Sand W, Willscher S (2013). Biomining: metal recovery from ores with microorganisms. Geobiotechnol i Adv Biochem Eng.

[CR156] Schuerger AC, Nicholson WL (2016). Twenty species of hypobarophilic bacteria recovered from diverse soils exhibit growth under simulated martian conditions at 0.7 kPa. Astrobiology.

[CR157] Schwandt C, Hamilton JA, Fray DJ, Crawford IA (2012). The production of oxygen and metal from lunar regolith. Planet Space Sci.

[CR158] Schwendner P, Schuerger AC (2020). Exploring microbial activity in low-pressure environments. Curr Issues Mol Biol.

[CR159] Senatore G, Mastroleo F, Leys N, Mauriello G (2018). Effect of microgravity and space radiation on microbes. Fut Microbiol.

[CR160] Singh S (2020). Biosorption of heavy metals by cyanobacteria: potential of live and dead cells in bioremediation. Microbial bioremediation and biodegradation.

[CR161] Singh S, Cameotra SS, Sukla LB, Pradhan N, Panda S, Mishra BK (2015). Anaerobic bioleaching by acidophilic bacterial strains. Environmental microbial biotechnology.

[CR162] Sivakumar V, Neelakantan R, Santosh M (2017). Lunar surface mineralogy using hyperspectral data: Implications for primordial crust in the Earth–Moon system. Geosci Front.

[CR163] Slenzka K, Kempf J (2010) Bio-ISRU concepts using microorganisms to release O_2_ and H_2_ on Moon and Mars. In: 38th COSPAR Scientific Assembly 2010

[CR164] Srichandan H, Mohapatra RK, Parhi PK, Mishra S (2019). Bioleaching approach for extraction of metal values from secondary solid wastes: a critical review. Hydrometallurgy.

[CR165] Steenstra ES, Agmon N, Berndt J, Klemme S, Matveev S, Van Westrenen W (2018). Depletion of potassium and sodium in mantles of Mars, Moon and Vesta by core formation. Sci Rep.

[CR166] Steenstra ES, Berndt J, Klemme S, Rohrbach A, Bullock ES, van Westrenen W (2020). An experimental assessment of the potential of sulfide saturation of the source regions of eucrites and angrites: Implications for asteroidal models of core formation, late accretion and volatile element depletions. Geochim Cosmochim Acta.

[CR167] Stern SA (1999). The lunar atmosphere: history, status, current problems, and context. Rev Geophys.

[CR168] Stevens AH, Childers D, Fox-powell M, Nicholson N, Jhoti E, Cockell CS (2019). Growth, viability, and death of planktonic and biofilm *Sphingomonas desiccabilis* in simulated martian brines. Astrobiology.

[CR169] Tait AW, Gagen EJ, Wilson SA, Tomkins AG, Southam G (2017). Microbial populations of stony meteorites: substrate controls on first colonizers. Front Microbiol.

[CR170] Taylor GJ (2013). The bulk composition of Mars. Chem Erde.

[CR171] Taylor SR, Taylor GJ, Taylor LA (2006). The moon: a taylor perspective. Geochim Cosmochim Acta.

[CR172] Tsuchiyama A, Uesugi M, Matsushima T, Michikami T, Kadono T, Nakamura T (2011). Evolution of Itokawa regoliththree-dimensional structure of hayabusa samples: origin and evolution of itokawa regolith. Science.

[CR173] Turner J, Anderson P, Lachlan-Cope T, Colwell S, Phillips T, Kirchgaessner A (2009). Record low surface air temperature at Vostok station, Antarctica. J Geophys Res Atmos.

[CR174] Ulrich N, Nagler K, Laue M, Cockell CS, Setlow P, Id RM (2018) Experimental studies addressing the longevity of *Bacillus subtilis* spores—The first data from a 500-year experiment. 1–14. 10.1371/journal.pone.0208425 PMC6279046 30513104

[CR175] Valix M (2017). Bioleaching of electronic waste: milestones and challenges.

[CR176] Verseux C (2020). Bacterial growth at low pressure : a short review. Front Astron Sp Sci.

[CR177] Verseux C, Baqué M, Lehto K, De Vera JPP, Rothschild LJ, Billi D (2016). Sustainable life support on Mars—the potential roles of cyanobacteria. Int J Astrobiol.

[CR178] Verseux C, Heinicke C, Ramalho T, Determann J, Smagin M, Avila M (2021). A low-pressure, N2/CO2 atmosphere is suitable for cyanobacterium-based life-support systems on Mars. Front Microbiol.

[CR179] Volger R, Pettersson GM, Brouns SJJ, Rothschild LJ, Cowley A, Lehner BAE (2020). Mining moon and mars with microbes: biological approaches to extract iron from Lunar and Martian regolith. Planet Space Sci.

[CR180] Volponi M, Lasseur C (2020). Considerations on life support systems for interstellar travel: a regenerative story. Acta Fut.

[CR181] Wadsworth J, Cockell CS (2017). Perchlorates on Mars enhance the bacteriocidal effects of UV light. Sci Rep.

[CR182] Wang Z, Becker H (2017). Chalcophile elements in Martian meteorites indicate low sulfur content in the Martian interior and a volatile element-depleted late veneer. Earth Planet Sci Lett.

[CR183] Williams JP, Paige DA, Greenhagen BT, Sefton-Nash E (2017). The global surface temperatures of the moon as measured by the diviner lunar radiometer experiment. Icarus.

[CR184] Wittenberg LJ, Santarius JF, Kulcinski GL (1986). Lunar source of 3 He for commercial fusion power. Fusion Technol.

[CR185] Yamada-Onodera K, Mukumoto H, Katsuyama Y, Tani Y (2002). Degradation of long-chain alkanes by a polyethylene-degrading fungus *Penicillium Simplicissimum* YK. Enzyme Microb Technol.

[CR186] Yen AS, Gellert R, Schröder C, Morris RV, Bell JF, Knudson AT (2005). An integrated view of the chemistry and mineralogy of martian soils. Nature.

[CR187] Yoshizaki T, McDonough WF (2020). The composition of Mars. Geochim Cosmochim Acta.

[CR188] Yoshizaki T, McDonough WF (2021). Earth and mars—distinct inner solar system products. Chem Erde.

[CR189] Yu R, Liu J, Tan J, Zeng W, Shi L, Gu G (2014). Effect of pH values on the extracellular polysaccharide secreted by *Acidithiobacillus ferrooxidans* during chalcopyrite bioleaching. Int J Miner Metall Mater.

[CR190] Zea L (2015) Phenotypic and gene expression responses of *E. coli* to antibiotics during spaceflight. Doctoral dissertation, Univ. Colorado. Boulder, Colorado, USA. https://www.colorado.edu/faculty/zea-luis/sites/default/files/attached-files/zea_-_thesis_-_published.pdf.

[CR191] Zea L, Prasad N, Levy SE, Stodieck L, Jones A, Shrestha S (2016). A molecular genetic basis explaining altered bacterial behavior in space. PLoS ONE.

[CR192] Zea L, Larsen M, Estante F, Qvortrup K, Moeller R, de Oliveira SD (2017). Phenotypic changes exhibited by *E. coli* cultured in space. Front Microbiol.

[CR193] Zea L, Nisar Z, Rubin P, Luo J, Mcbride SA, Moeller R (2018). Design of a spaceflight biofilm experiment. Acta Astronaut.

[CR194] Zea L, Mclean RJC, Rook TA, Angle G, Carter DL, Delegard A (2020). Potential biofilm control strategies for extended spaceflight missions. Biofilm.

[CR195] Zeitlin C, Hassler DM, Cucinotta FA, Ehresmann B, Wimmer-Schweingruber RF, Brinza DE, Kang S, Weigle G, Böttcher S, Böhm E, Burmeister S, Guo J, Köhler J, Martin C, Posner A, Rafkin S, Reitz G (2013). Measurements of energetic particle radiation in transit to Mars on the Mars science laboratory. Science.

[CR196] Zhang S, Wimmer-Schweingruber RF, Yu J, Wang CC, Fu Q, Zou Y (2020). First measurements of the radiation dose on the lunar surface. Sci Adv.

